# Endobronchial removal of the peripherally located foreign body with the ultrathin bronchoscopy and ultrathin cryoprobe guided by a manual navigating method: A case report

**DOI:** 10.1097/MD.0000000000031903

**Published:** 2022-12-02

**Authors:** Mingli Yuan, Yang Xiao, Fang Ni, Wen Yin, Yi Hu

**Affiliations:** a Department of Pulmonary and Critical Care Medicine, Central Hospital of Wuhan, Tongji Medical College, Huazhong University of Science and Technology, Wuhan, China.

**Keywords:** foreign body aspiration, manual navigation, ultrathin bronchoscope (UTB), ultrathin cryoprobe

## Abstract

The bronchoscope is a preferential method used to remove airway foreign bodies, but for those located in the distal lumen of bronchus with long-time retention, how to remove them remains an intractable problem.

**Patient concerns:**

A 57-year-old male presented with 2-week history of intermittent hemoptysis. Chest CT upon admission revealed a high-density opacity incarcerated in the distal basal segment of the left lower lobe, along with obstructive pneumonia.

**Diagnoses:**

The patient was diagnosed as foreign body aspiration.

**Interventions:**

We firstly used a manual navigating method to draw a bronchoscopic map according to the thin-section CT. Then we adopted ultrathin bronchoscope (UTB) to remove the peripherally located foreign body.

**Outcomes:**

UTB successfully found the foreign body incarcerated in LB10ciiβ under the guidance of manual navigation, but it was too tender to be extracted completely by forceps, and it was even pushed further away. Then 1.1 mm ultrathin cryoprobe was used, with an activation time of 4 seconds, the chili was frozen and completely removed.

**Lessons:**

This first combined application of manual navigating method, UTB and ultrathin cryoprobe, successfully extracted foreign bodies lodged in the distal airways and thus avoided thoracic surgery.

## 1. Introduction

Foreign body aspiration in adults is characterized as chronic aspiration, which is defined as chocking history of more than 1 month or no definite chocking history,^[1]^ delayed diagnosis,^[2]^ and peripherally located foreign bodies.^[2]^ It may present as focal high-density opacity, focal bronchiectasis, recurrent pneumonia, or lung collapse in the affected lobe in CT images.^[3]^ The bronchoscope is a preferential method used to diagnose airway foreign body aspiration directly, and when combined with forceps, loops or baskets, it achieves a high success rate in removing foreign bodies.^[1]^ Cryotherapy, which is widely used in diagnosing and treatment of lung tumors,^[4–6]^ also plays an important role in the cases of hyperplastic granulation tissue surrounding the foreign body with long-time retention in the airway.^[1]^

For peripherally located foreign bodies, which could not be observed by conventional bronchoscope, surgical operation seemed to be the only solution in the past. However, as the development of interventional bronchoscopy, with the help of bronchial navigation technique, ultrathin bronchoscope (UTB), which has been reported for the diagnosis of peripheral pulmonary lesions,^[7]^ and ultrathin accessory tools, peripherally located foreign bodies can be removed and thus avoid thoracic surgery.

We herein introduce an economical, but highly efficient navigation method, the manual navigating method, and report a case of adult chronic foreign body aspiration successfully treated by UTB and ultrathin cryoprobe guided by this navigating method.

## 2. Case report

**Figure 1.**
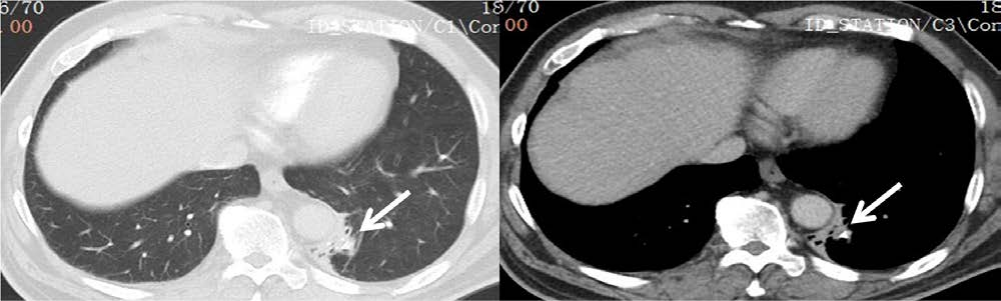
Chest CT upon admission revealed a high-density opacity incarcerated in the distal basal segment of left lower lobe (arrow), along with obstructive pneumonia.

A 57-year-old male presented with 2-week history of intermittent hemoptysis. He had a smoking history of 20 pack-years, no history of alcohol intake and no definite choking history. His vital signs were normal and systemic examination was unremarkable. Chest CT upon admission revealed a high-density opacity incarcerated in the distal basal segment of the left lower lobe, along with obstructive pneumonia (Fig. 1).

**Figure 2.**
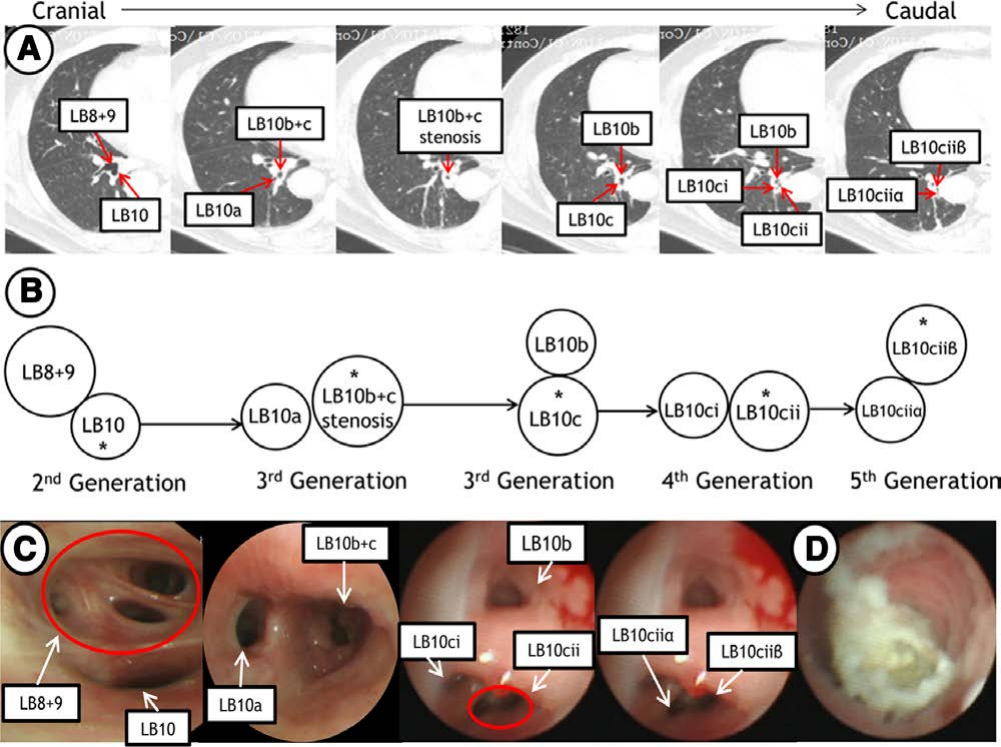
(A and B) Schematic drawing of left lower basal segment corresponding to CT images via manual navigating method. (A) Flipped axial CT images around the vertical axis to correlate with bronchoscope movement in a cranial-caudal direction. (B) Traced the bronchial branch in which the lesion was located, and recorded with a bronchial opening sketch at every bifurcation point. (C) Bronchoscopic images were consistent with simulated map. (D) The foreign body was located in LB10ciiβ, with obvious granulation tissue and purulent secretion.

Before the bronchoscopy, we used a manual navigating method to locate the lesion. Firstly, we read his thin-section CT images and a 3-dimensional CT image reconstruction to identify which bronchial branch the lesion was located by. Then, flipped axial CT images around the vertical axis to match the reverse bronchoscopic view (Fig. 2A), rolled CT images continuously and recorded every bifurcation point with a bronchial opening sketch from the second generation bronchus to the leading bronchus which the lesion was located by Fig. 2B. Thus, a simulated image of endoscopic image was made. The lesion was in LB10ciiβ according to our simulated image. The patient received general anesthesia with laryngeal mask airway. Firstly, the therapeutic bronchoscope (1TQ290, Olympus, 5.9 mm outer diameter) failed in finding the foreign body, because it could not proceed further into LB10 due to the stenosis of the lumen. We then switched to UTB (BF-XP260, Olympus) with 2.8 mm outer diameter and 1.2 mm working channel. The endoscope approached according to the simulated image, and the foreign body was indeed found incarcerated in the lumen of LB10ciiβ, with obvious granulation tissue and purulent secretion (Fig. 2C and D). Forceps was tried to clamp and remove the foreign body, but failed due to blockage by the remarkable granulation tissue. Then we switched to the therapeutic bronchoscope (1TQ290, Olympus, 5.9 mm outer diameter), and removed the granulation tissue in the proximal portion of LB10b + c by the high-frequency electrocautery probe. Again, we used UTB and forceps to clamp the foreign body, but the foreign body was too tender to be extracted in a whole piece. After several times of clamping, the foreign body was gradually extracted, and it turned out to be fragments of a tiny chili. We then examined the airway and found LB10c seeming clear under the bronchoscope.

**Figure 3.**
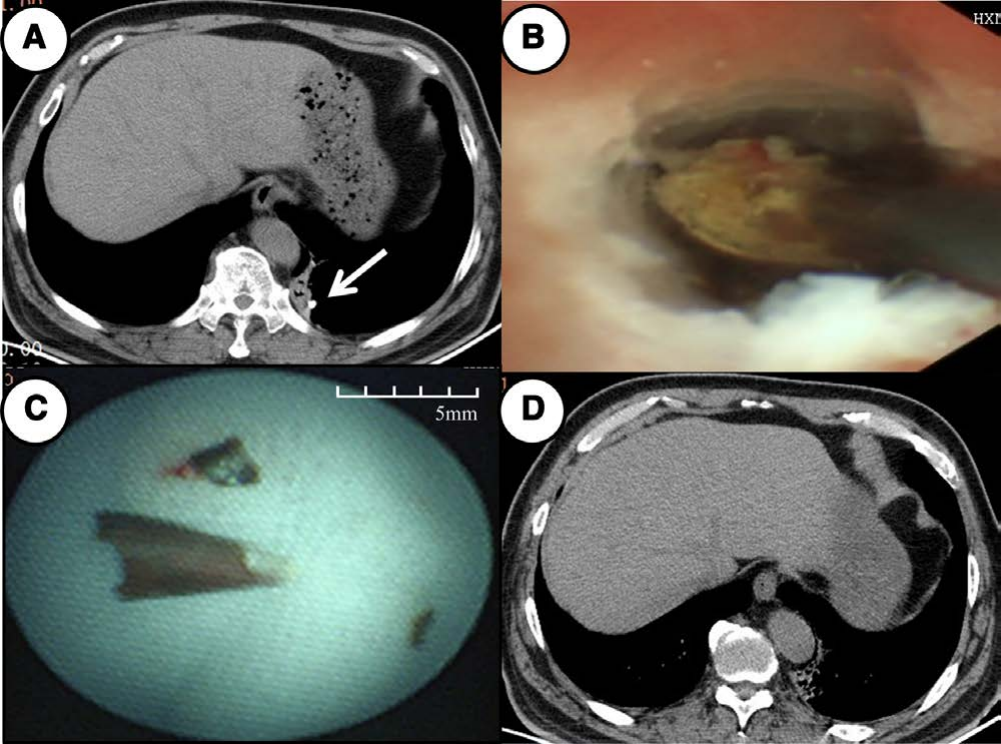
(A) Reexamined CT images indicated that the foreign body was pushed further away (arrow). (B) The cryoprobe was inserted into ultrathin bronchoscope (UTB), keeping the probe against the foreign body, and freezing. (C) The remaining chili was completely extracted. (D) Reexamined CT images confirmed the completely removal of the foreign body.

However, reexamined CT indicated that the residual foreign body was pushed further away (Fig. 3A). Then the second bronchoscopy was performed under general anesthesia with tracheal intubation. UTB revealed the remaining chili was still incarcerated in the distal LB10ciiβ. To avoid pushing away or crushing the foreign body, we adopted an ultrathin cryoprobe with 1.1 mm outer diameter (ERBECRYO2, ERBE). As shown in Figure 3B, the cryoprobe was inserted into UTB, keeping the probe against the foreign body, and freezing for 4 seconds, and then it was removed with the UTB together out of the airway. The foreign body was frozen, and after twice freezing, the remaining chili was completely extracted (Fig. 3C). Reexamined CT confirmed the completely removal of the foreign body (Fig. 3D).

## 3. Discussion

Previous studies have demonstrated a relatively high prevalence of chronic aspiration secondary to foreign bodies in adults, and the most common foreign bodies were organic bone and vegetables.^[1,8]^ Adult patients with chronic aspiration often have no apparent symptoms except for coughing as the foreign body enters the airway, and some even have no definite history of choking. Thus, recurrent obstructive pneumonia in patients with alcoholism, advanced age, and impaired swallowing reflex should be caution of foreign body aspiration.

In adults with chronic aspiration, foreign bodies are often lodged in more distal bronchial tree,^[2]^ and it’s important to know how to get to the site where foreign bodies located before bronchoscopy. There have been various navigation technologies, such as electromagnetic navigational bronchoscopy and virtual bronchoscopic navigation. Virtual bronchoscopic navigation can improve the sensitivity and specificity of the foreign body finding up to 92.3% and 85.7%, respectively.^[9]^ However, these advanced navigation systems are not available in all bronchoscopy centers. The manual navigating method, which has not been widely applied, is not only economical, but also brings a high navigation success rate and an increase diagnostic yield in peripheral pulmonary lesions.^[10,11]^ In the case of the deformation and stenosis of the airways due to the significant granulation tissue in our case, we might not find out the lesion successfully without keeping the bronchial map at hand. To the best of our knowledge, its utility in removal of foreign bodies has not been reported.

Flexible conventional bronchoscopy exhibits excellent efficacy in removal of foreign bodies, with a 1-step success rate of 90%,^[1]^ but in such situations: foreign bodies located in the distal lumen of bronchus with long-time retention, large or hard foreign bodies penetrating through the bronchial wall, osseous or metallic foreign bodies being close to large vessels, thoracic surgery remains as the last choice.^[12]^ Thanks to the UTB, the foreign body located in the fifth generation bronchus with significant granulation tissue in our case, might undergo thoracic surgery if it could not be removed by bronchoscopy. UTB is defined as having an outer diameter ≤3.5 mm, and most conventional UTBs are equipped with a working channel with an inner diameter of 1.2 mm.^[7]^ The UTB has good maneuverability for passing through the small bronchi as well as good accessibility to peripheral lung lesions, and exhibits a high diagnostic value in sampling the peripheral lesions.^[7]^ However, its utility in removal of foreign bodies has been rarely reported.

The challenge of the UTB for peripherally lesions is that it allows the use of only mini-forceps < 1.2 mm in diameter, which could only obtain limited specimens because of the mini-size of clamp body and insufficient space in distal airway for opening the forceps. While 1.1 mm ultrathin cryoprobe can easily bend and extend to the distal bronchus, and harvest several-fold amount of specimens by the movement of freezing and pulling out the probe, thus it is feasible, and has a high diagnostic yield in the peripheral pulmonary lesions.^[6,13]^ Cryotherapy is also used in removal of foreign bodies, mainly used to eliminate granulation tissue surrounding the foreign body.^[1]^ Mini-forceps was firstly tried in our case, and sometimes it was hard to open in such a small space or to clamp for that the chili stuck to the bronchial wall. Moreover, the foreign body was so tender that it broke into pieces after several times of clamping, and was even pushed further away. The ultrathin cryoprobe, which perfectly made up for these shortcomings of forceps, had low requirements for working space, directly froze the whole foreign body without crushing, and successfully removed the chili completely. This is the first report of ultrathin cryoprobe in removal of foreign bodies.

Our case report firstly highlights the usefulness of the manual navigating method, UTB and ultrathin cryoprobe in extracting foreign bodies lodged in the distal airways, thus avoid thoracic surgery.

## Author contributions

**Conceptualization:** Mingli Yuan and Yi Hu.

**Data curation:** Yang Xiao, Fang Ni and Wen Yin.

**Writing – original draft:** Mingli Yuan and Yang Xiao.

**Writing – review and editing:** Yi Hu.

## References

[1] Fang YF Hsieh MH Chung FT. Flexible bronchoscopy with multiple modalities for foreign body removal in adults. PLoS One. 2015;10:e0118993. 25768933 10.1371/journal.pone.0118993 PMC4358882

[2] Baharloo F Veyckemans F Francis C. Tracheobronchial foreign bodies: presentation and management in children and adults. Chest. 1999;115:1357–62. 10334153 10.1378/chest.115.5.1357

[3] Zissin R Shapiro-Feinberg M Rozenman J. CT findings of the chest in adults with aspirated foreign bodies. Eur Radiol. 2001;11:606–11. 11354755 10.1007/s003300000619

[4] Vergnon JM Huber RM Moghissi K. Place of cryotherapy, brachytherapy and photodynamic therapy in therapeutic bronchoscopy of lung cancers. Eur Respir J. 2006;28:200–18. 16816349 10.1183/09031936.06.00014006

[5] Hetzel J Eberhardt R Herth FJ. Cryobiopsy increases the diagnostic yield of endobronchial biopsy: a multicentre trial. Eur Respir J. 2012;39:685–90. 21852332 10.1183/09031936.00033011

[6] Jiang S Liu X Chen J. A pilot study of the ultrathin cryoprobe in the diagnosis of peripheral pulmonary ground-glass opacity lesions. Transl Lung Cancer Res. 2020;9:1963–73. 33209616 10.21037/tlcr-20-957 PMC7653104

[7] Oki M Saka H. Diagnostic value of ultrathin bronchoscopy in peripheral pulmonary lesions: a narrative review. J Thor Dis. 2020;12:7675–82. 10.21037/jtd-2020-abpd-001 PMC7797850 33447460

[8] Casalini AG Majori M Anghinolfi M. Foreign body aspiration in adults and in children: advantages and consequences of a dedicated protocol in our 30-year experience. J Bronchol Interv Pulmonol. 2013;20:313–21. 10.1097/LBR.0000000000000024 24162114

[9] Bhat KV Hegde JS Nagalotimath US. Evaluation of computed tomography virtual bronchoscopy in paediatric tracheobronchial foreign body aspiration. J Laryngol Otol. 2010;124:875–9. 20426892 10.1017/S0022215110000769

[10] Kho SS Nyanti LE Chai CS. Feasibility of manual bronchial branch reading technique in navigating conventional rEBUS bronchoscopy in the evaluation of peripheral pulmonary lesion. Clin Resp J. 2021;15:595–603. 10.1111/crj.13297 33113256

[11] Zhang L Tong R Wang J. Improvements to bronchoscopic brushing with a manual mapping method: a three-year experience of 1143 cases. Thor Cancer. 2016;7:72–9. 10.1111/1759-7714.12279 PMC4718127 26816541

[12] Huang H Seif MM Ren J. Endobronchial removal of the high-risk osseous foreign bodies with evaluation and planning by virtual navigation system. Resp Med Case Rep. 2019;28:100952. 10.1016/j.rmcr.2019.100952 PMC6831871 31709141

[13] Kho SS Chai CS Nyanti LE. Combination of 1.1 mm flexible cryoprobe with conventional guide sheath and therapeutic bronchoscope in biopsy of apical upper lobe solitary pulmonary nodule. BMC Pulm Med. 2020;20:158. 32493437 10.1186/s12890-020-01199-3 PMC7269002

